# Cutaneous metastasis as a first presentation of lung carcinoma: a case series

**DOI:** 10.1186/s13256-023-04029-2

**Published:** 2023-07-23

**Authors:** Saida Sakhri, Ines Zemni, Mohamed Ali Ayadi, Lamia Naija, Nadia Boujelbene, Tarek Ben Dhiab

**Affiliations:** 1 Department of Surgical Oncology,, Salah Azaiez Institute, Faculty of Medicine of Tunis, University of Tunis El Manar, Tunis, Tunisia; 2 LMBA (LR03ES03), Sciences Faculty of Tunis, University of Tunis El Manar, Tunis, Tunisia; 3 Department of Pathology, Salah Azaiez Institute, Faculty of Medicine of Tunis, University of Tunis El Manar, Tunis, Tunisia

**Keywords:** Skin metastasis, Lung carcinoma, Pathology, Immunohistochemistry, Prognosis

## Abstract

**Introduction:**

Cutaneous metastases (CM) revealing lung carcinoma are extremely rare, accounting for 0.8%. The diagnosis is guided by histology and immunohistochemistry. Treatment is palliative. The prognosis is poor.

**Case presentation:**

This is a retrospective study of the available clinical and histological records of four North African patients with CM revealing lung cancer treated at our institute between 2004 and 2010. Three men and one woman were registered. The mean age was 54.5 years (38–74 years). Two patients had primary adenocarcinoma, one patient had small cell carcinoma and one had squamous cell carcinoma. Treatment was based on chemotherapy in two cases and antalgic radiotherapy in two cases, one patient underwent surgical resection as the lesion was infected. The overall survival after diagnosis was between one and four months.

**Conclusions:**

A skin nodule can be the first symptom revealing lung cancer. A rare clinical presentation that should not be taken for a benign nodule, the biopsy and histological study with immunohistochemistry confirm the diagnosis.

## Introduction

Cutaneous metastasis (CM) from primary visceral malignancy is an uncommon entity. The incidence is between 1 and 12%. skin metastasis from lung carcinoma account for 3.4%. Generally it occurs at the terminal stage of the disease, but it may rarely reveal the illness in 0.8% [1, 2]. We aim to report our experience and discuss diagnoses and therapeutic management with a review of the literature.

## Case presentation

**Fig. 1:**
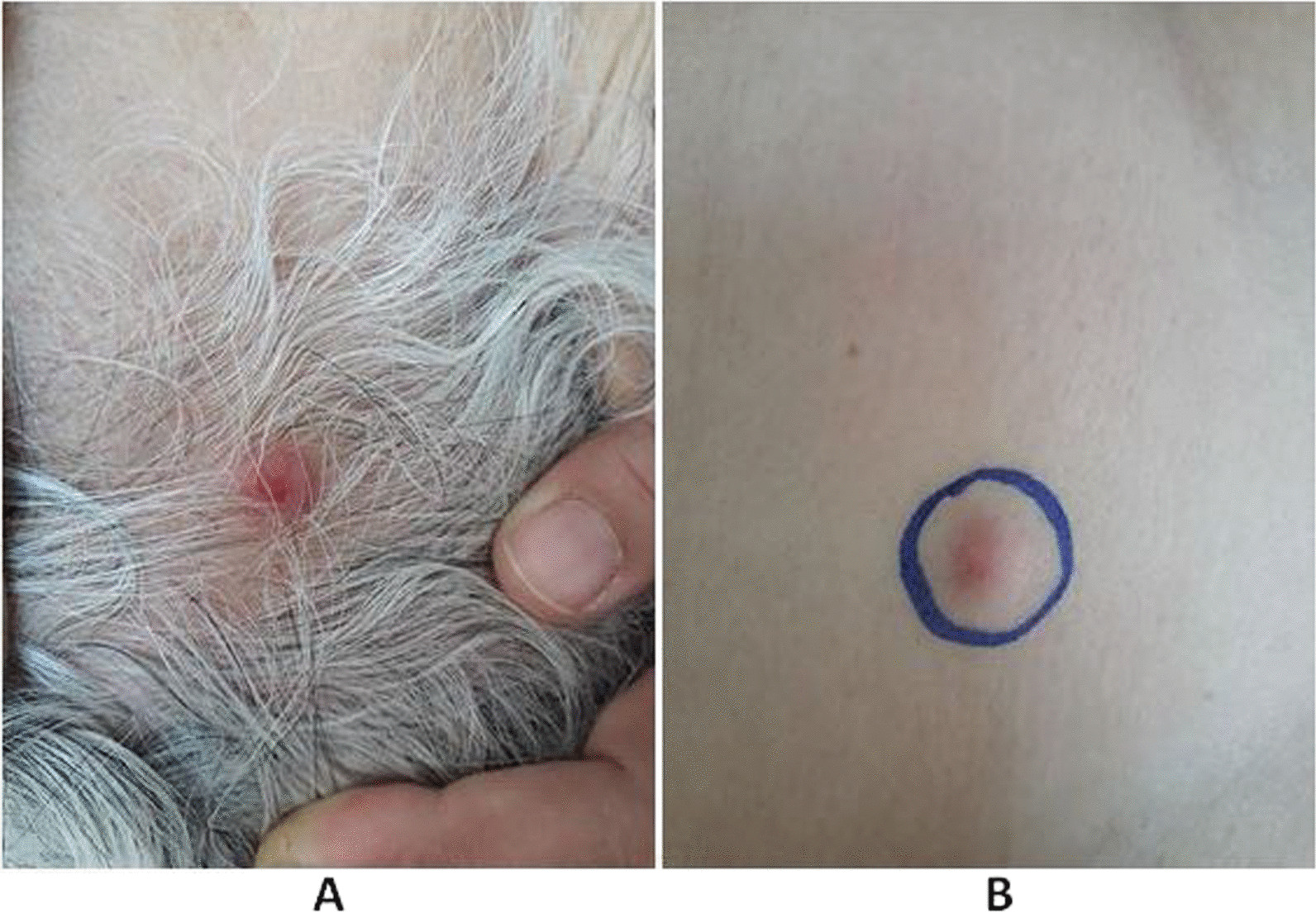
Hard nodules on the scalp (**A**) and thorax (**B**): nodule with telangiectasia (blue circle)

**Fig. 2:**
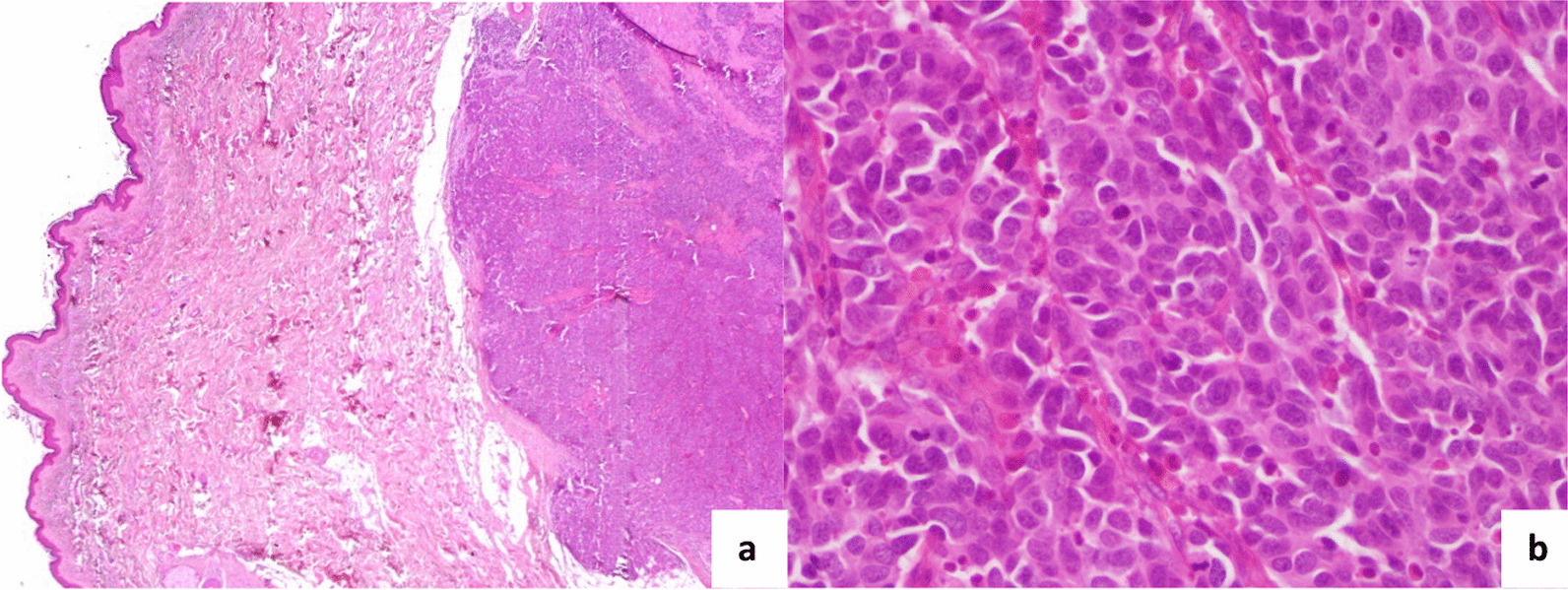
Cutaneous metastasis of a lung adenocarcinoma. **a** Poorly differentiated adenocarcinoma within dermis and hypodermis (Haematoxylin–eosin, 40 × magnification) **b** sheets and clusters of atypical cells with numerous mitosis (Haematoxylin–eosin, 400 × magnification)

**Fig. 3:**
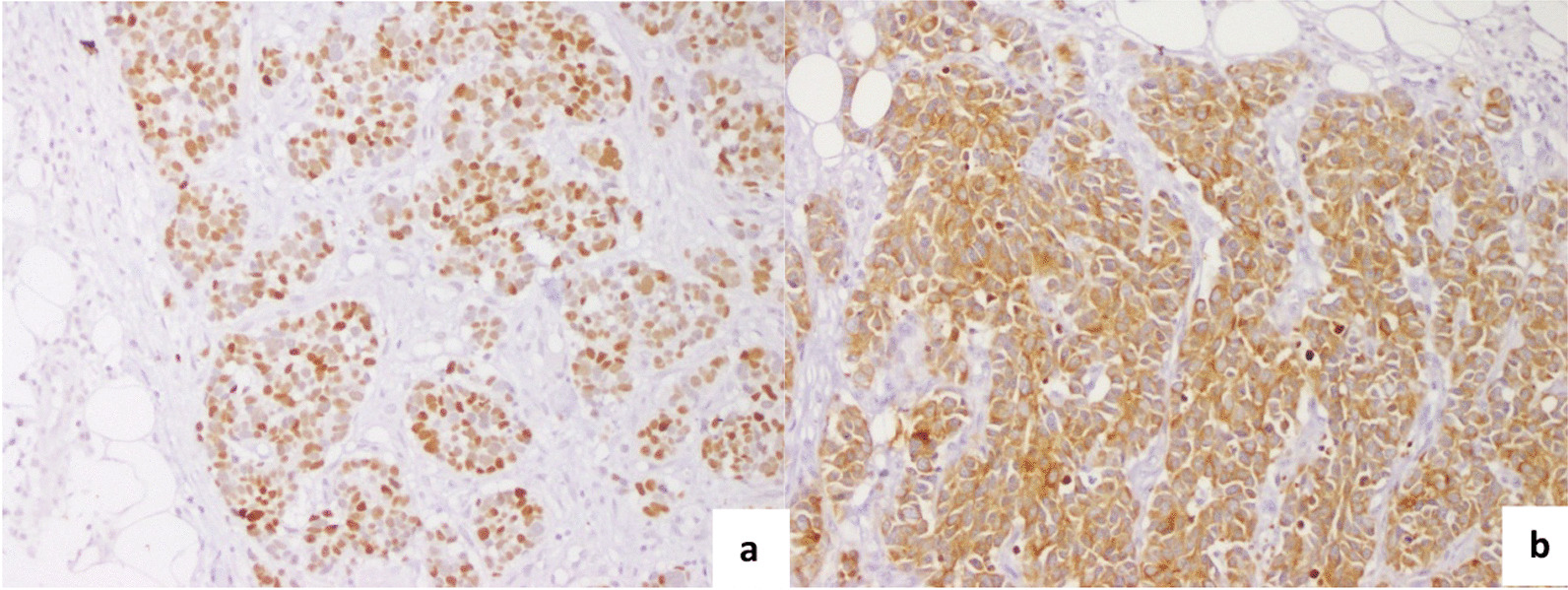
Cutaneous metastasis of lung adenocarcinoma Immunohistochemistry findings. **a** Diffuse TTF1 and **b** CK7 expression (200 × magnification)

We reported four North African cases of cutaneous metastases revealing lung cancer diagnosed in the surgical department of the Salah Azaiez Institute in Tunisia. The mean age was 54.5 years (38–74 years). The sex ratio was 3/1. All the male patients were active smoking; however, the female patient was passive smoking. Skin metastasis was located in the scalp (2 cases), thorax (one case) and thoraco-abdominal in one case (Fig. 1). The clinical presentation was subcutaneous hardness in 3 cases, and in one case the lesion was infected and ulcerated. It was unique in two cases. The mean tumor size was 15 mm (3 to 20 mm). Biopsy revealed adenocarcinoma in two cases, small cell carcinoma in one case, and squamous cell carcinoma (Fig. 2). Immunohistochemistry (IHC) showed positivity for CK7, TTF1, and negativity for CK20, CK5/6, pointing to a primary lung carcinoma (Fig. 3). We performed chest radiography for all patients, showing a suspicious lung lesion. Body scan and endoscopy showed a stage IV lung tumor in all cases. Patient and tumor characteristics are presented in Table 1.

**Table 1:** The characteristics of primitive lung tumor

Case	Age	Gender	Histological type	Classification
1	56	Male	Adenocarcinoma	T3N1M1 (bone)
2	50	Male	Small cell carcinoma	T4N3M1 (liver, bone)
3	38	Male	Squamous cell carcinoma	T4N1M1 (brain)
4	74	Female	Adenocarcinoma	T4N3M1 (bone)

Treatment was based on palliative platinum-based chemotherapy (cisplatin) of 3 to 6 courses in two cases, and analgesic radiotherapy in two cases, surgery was performed in one case due to the infected lesion. The average follow-up was 3 months. The survival rate was between one and four months.

## Discussion

Dissemination of visceral malignancies to the skin is rare and usually occurs in advanced diseases. However, it can be a sign of a clinically silent malignancy [3, 4]. Skin metastases can occur anywhere in the body, but usually in close proximity to the primary tumor. In fact, the literature shows that lung cancer metastases primarily involve the chest, followed by the scalp and arms. Babacan *et al*. found that the scalp is the most common site of skin metastases in 54% of cases and the arms are the least common site in 0.2% of cases. The reason why scalp metastases are more common may be due to rich blood supply [2, 5].

Cutaneous metastases of lung cancer do not have a characteristic presentation. Wang, Babacan, and Abdeen described these metastases as nodular, mobile or fixed, indurated, painless, varying in size (5 to 60 mm), and often covered by normal skin, they may be solitary or multiple. Other clinical aspects include ulcerations, erysipelasis, bullae, and vascular tumors with telangiectasias [2, 4, 6].

The diagnosis is often based on clinical information; look for respiratory and systemic complaints or a history of smoking. The first complementary examination is the chest X-ray. CT scan remains the best way to evaluate the local extension. However, the diagnosis is confirmed by biopsy and histological examination, which show that they are often poorly differentiated. CM occurs after the invasion of the lymphovascular system and is often limited to the dermis and subcutaneous layer [5, 7].

All histological subtypes can give rise to CM. Abdeen and YU reported that adenocarcinoma is the most common type, followed by squamous, small, and large cell carcinoma. Other studies have shown that large cell carcinoma is the most common subtype and squamous cell carcinoma is the least common. Olso YI found that non-small cell carcinoma occurs primarily in women and non-smokers [7, 8].

Immunohistochemistry remains the gold standard confirmatory method using specific markers. The positivity of TTF1 and CK7 suggests a primary lung tumor, other markers are useful such as antithyroid transcription factor and CK-20 [8, 9].

CM in lung cancer patients is associated with an aggressive tumor. Usually, only palliative chemotherapy is offered (combinations of cisplatin and etoposide or cyclophosphamide, adriamycin, and vincristine). But the response is minimal because of the poor blood supply to the skin. Radiation therapy is usually indicated for pain and bleeding [6, 10].

Treatment of the solitary lesion can be a combination of surgery and chemotherapy, it is shown that resection of the skin lesion offers a better survival with a gain of 3 months. But in all cases, this indicates an advanced stage of cancer. The prognosis remains poor and the survival rate after diagnosis varies between three and five months. Prognostic factors are small cell carcinoma, undifferentiated tumor, multiple metastatic lesions, and other distant metastases [2, 10, 11].

## Conclusion

CM revealing lung carcinoma is extremely rare. It may be confused with other benign lesions such as vascular, erysipeloid, and bullous forms. Therefore, skin biopsy and histological examination, especially immunohistochemistry, are helpful in identifying the histological type of primary cancer. Treatment is palliative and based on chemotherapy, but the prognosis remains poor.

## Data Availability

Data supporting our findings were taken from the patient’s folder.
